# Biosynthesis-based metabolomics analysis reveals chemical diversity between two *Salvia* species

**DOI:** 10.3389/fpls.2025.1613313

**Published:** 2025-07-04

**Authors:** Feiyan Wang, Chenyi Li, Haizheng Yu, Dongfeng Yang, Ji Ye, Lei Zhang

**Affiliations:** ^1^ Innovative Drug Research Center, College of Life Sciences and Medicine, Key Laboratory of Plant Secondary Metabolism and Regulation of Zhejiang Province, Zhejiang Sci-Tech University, Hangzhou, China; ^2^ State Key Laboratory of Genetic Engineering, Collaborative Innovation Center of Genetics and Development, Department of Biochemistry, Institute of Plant Biology, School of Life Sciences, Fudan University, Shanghai, China; ^3^ Department of Pharmaceutical Botany, School of Pharmacy, Naval Medical University, Shanghai, China

**Keywords:** spatial metabolomics, chemical diversity, volatile components, phenolic acids, carnosic acid, *Salvia officinalis*, *Salvia miltiorrhiza* Bunge

## Abstract

*Salvia officinalis* is an important dietary supplement that is widely used as a flavor regulator and plays an important role in the prevention and treatment of neurodegenerative diseases. *Salvia miltiorrhiza* Bunge is a famous Chinese herbal medicine for treating cardiovascular diseases. Secondary metabolites with diverse structures endow the two species with various edible and medicinal values. However, the differences in secondary metabolites between the leaves of *S. officinalis* and *S. miltiorrhiza* are still unclear. Herein, FlavourSpec^®^ combined with spatial metabolomics was used to explore the distribution patterns of secondary metabolites including volatile and non-volatile components. The results indicated that the chemical compositions of the two *Salvia* species were significantly different. Specifically, *S. miltiorrhiza* Bunge contained high levels of phenolic acid components with a furan ring that can hardly be detected in *S. officinalis.* The volatile small molecules as well as carnosic acid and its derivatives were found to be major components of *S. officinalis* leaves. Because of the long-term exposure of leaves to ultraviolet radiation and the same environmental stress, carnosic acid and its derivatives exhibit widespread distribution characteristics in *S. officinalis* leaves. The work explored the similarities and differences in secondary metabolites of *S. officinalis* and *S. miltiorrhiza* Bunge, providing not only the material basis to develop the application value in dietary nutrition, but also a theoretical foundation for the development and utilization of medicinal resources of *Salvia*.

## Highlights

- The method to detect the spatial distribution of chemicals in-situ was developed.
- Carnosic acid and carnosol were widely distributed in *S. officinalis* leaves.
- Flavourspec® was first used to explore the aromatic components in *Salvia* species.
- *S.officinalis* was found as perfect material to extract plant essential oils.
- The leaves of *S.militiorhiza* were rich in phenolic acid polymer with furan rings.

## 1 Introduction

*Salvia officinalis* is an important dietary supplement that not only is widely used as a flavor regulator in food processing but also plays an important role in the prevention and treatment of neurodegenerative diseases (Ghorbani and Esmaeilizadeh, 2017; Uță et al., 2021). Carnosic acid and its derivatives are reported as main active compounds in *S. officinalis* (Birtić et al., 2015). Moreover, it was found that *S. officinalis* is also rich in volatile components and can be used to treat diseases such as epilepsy, ulcers, rheumatism, and hyperglycemia (Koubaa et al., 2019). *S. miltiorrhiza* is a traditional Chinese herbal medicine and plays an important role in the prevention and treatment of cardiovascular diseases (Zhang et al., 2019). Tanshinones and phenolic acids were reported to have medicinal values (Wang and Peters, 2022). In recent years, volatile components in *S. miltiorrhiza* have also attracted widespread attention due to their significant functions; however, no systematic study has yet been performed to explore the difference between the chemical components of the two species (Li and Wang, 2009; Liang et al., 2009; Luo et al., 2014).

Flavourspec^®^ is a newly emerged gas-phase detection technology that combines gas chromatography (GC) with ion mobility spectrometry (IMS). It has high resolution and high sensitivity, is simple to operate, and performs rapid analysis compared with gas chromatography–mass spectrometry (GC-MS). The working principle of GC-MS is that helium or hydrogen gas carries volatile components through a chromatographic column under high-temperature conditions, and diverse interactions between chemicals and stationary phases lead to the separation of compositions (Beale et al., 2018). The mass spectrometer identifies the chemical composition and molecular structure of volatile components in the sample by measuring the mass-to-charge ratio (*m/z*) of atoms or molecules (Fiehn, 2016). However, an increasing number of studies indicated that temperature and other factors can obviously influence the chemical compositions of volatile components (D'Auria and Racioppi, 2015). For instance, as temperature increases, the content of 1,8-eucalyptus and camphor in the essential oils decreases, whereas the content of pinene increases (D'Auria and Racioppi, 2015). Flavourspec^®^ detects volatile components at normal temperature and pressure and proves to be a promising platform in the optimization of food processing methods and the selection of preservation methods (Yang et al., 2024).

Plants synthesize secondary metabolites with diverse structures to cope with changes in external environment and their own growth. Plant secondary metabolites typically have a wide range of biological activities and are important resources for developing new drugs. It is reported that structural diversity leads to diverse biological activities (Deng et al., 2023; Liu et al., 2023). Secondary metabolites typically exhibit significant structural diversity and tissue-specific distribution patterns due to the differences in stress and gene expression (Ramakrishna and Ravishankar, 2011). It is of great significance for their development and utilization to explore active natural products and elucidate their tissue-specific distribution patterns. With the development of spatial metabolomics, the spatial and temporal distribution properties of secondary metabolites can be elucidated. For instance, it was found that tanshinone was mainly distributed in the periderm of *S. miltiorrhiza* roots (Tong et al., 2022). In addition, the transcriptional landscape of main cell types in *Taxus wallichiana* leaves was also analyzed (Zhan et al., 2023), which was crucial for revealing cell-type-specific regulation of secondary metabolism. It is important to elucidate the spatiotemporal distribution characteristics of chemical components, which contribute to revealing the functional genes that regulate their synthesis for the quality control and resource development of medicinal plants. *S. miltiorrhiza* and *S. officinalis* are famous medicinal plants; the differences and similarities of secondary metabolites in their roots have been clarified (Hu et al., 2023). However, the accumulation patterns of secondary metabolites in leaves are still unclear. In this work, FlavourSpec^®^ was combined with spatial metabolomics to clarify the distribution pattern of volatile and non-volatile components in leaves. Desorption electrospray ionization mass spectrometry imaging (DESI-MSI) was further used to reveal the spatial distribution of secondary metabolites in *S. officinalis* to provide a basis for the secondary metabolism regulation network and functional food development of *S. officinalis* and *S. miltiorrhiza.*

## 2 Materials and methods

### 2.1 Materials and chemicals

Experimental materials were collected at the Shanghai National Forest Germplasm Resource Centre of Lamiaceae Plant (China). The samples were identified as *S. officinalis* and *S. miltiorrhiza* by associate professor Chenyi Li from the Institute of Plant Biology, School of Life Sciences, Fudan University. The Specimen Hall of Shanghai Botanical Garden was used to store the plant specimens.

Mature leaves with consistent physiological development stages and good phenotypic uniformity were selected as experimental materials. For the detection of volatile and non-volatile components, three biological replicates were independently set up for each treatment group. All the reference standards (purely analytical) including butanone, 2-butanone, 2-pentanone, 2-hexanone, 2-heptanone, 2-octanone, and 2-nonanone were purchased from Aladdin and used to calculate retention indices for the characterization of more volatile components. All the standards with purity above 98% including caffeic acid, rosmarinus acid, salvianolic acid B, lithospermic acid, and carnosic acid were acquired from BioBioPha (Yunnan, China). Formic acid was purchased from Fisher Scientific (Fair Lawn, NJ, USA), pure distilled water and leucine encephalin were obtained from Waters (Hong Kong, China), acetonitrile and methanol were purchased from Merck (Darmstadt, Germany), warfarin was purchased from Sigma-Aldrich (Madrid, Spain), and all reagents used in the spatial metabolome were LC-MS grade.

### 2.2 Sample preparation anddata collection for volatile compounds

The leaves of *S. miltiorrhiza* Bunge and *S. officinalis* (0.5 g) were taken into a 20-mL headspace injection bottle and incubated at a speed of 500 rpm for 20 min at 40°C. FlavourSpec^®^ was used to detect the chemical composition of volatile components. Each sample was measured three times in parallel. The conditions were as follows: MXT-5 capillary chromatography column (15 m × 0.53 mm, 1.0 μm); nitrogen was used as carrier gas and migratory gas; the injection needle temperature and column temperature were 85°C and 60°C, respectively; gradient elution was run for 30 min; the carrier gas flow was as follows: 0–2 min, 2 mL/min; 2–10 min, 2–20 mL/min; 10–20 min, 20–100 mL/min; and 20–30 min, 100–150 mL/min; migratory gas flow was 150 mL/min; the column temperature and IMS temperature were 60°C and 45°C, respectively; injection volume was 500 μL; and sampling in the non-shunt mode was used to collect data.

### 2.3 Sample preparation and data collection of LC-MS and DESI-MSI analysis

The leaves of *S. miltiorrhiza* Bunge and *S. officinalis* were crushed into homogeneous powders and sieved by a 40-mesh sieve. The prepared powders (0.01 g) were dissolved in 1 mL of 70% methanol (v/v) that contained warfarin (2 μg/mL); ultrasonic (53 kHz, 350 W) extraction was carried out for 60 min at greenhouse temperatures. The sample was then centrifuged at 12,000 rpm for 15 min to collect the supernatant. The vise was used and compressed with high pressure for probably 1 min to transfer metabolites of *S. officinalis* leaves to the PTFE membranes for DESI-MSI analysis.

The ACQUITY UPLC system (Waters) equipped with a binary pump, an autosampler, and a column compartment was combined with a Xevo G2-XS QTOF mass spectrometer (Waters) equipped with an electrospray ionization source to collect the non-volatile components in both positive- and negative-ion modes. An ACQUITY UPLC T3 column (2.1 mm × 100 mm, 1.8 µm) was used to separate the sample components at constant temperature (40°C). The 0.1% formic acid in water (solvent A) and in acetonitrile (solvent B) was taken as the mobile phase for gradient elusion. The elution gradient was as follows: 2%–20% B over 0–7 min, 20%–22% B over 7–11 min, 22%–60% B over 11–20 min, 60%–65% B over 20–25 min, 65%–65% B over 25–28 min, 65%–95% B over 28–30 min, 95%–95% B over 30–33 min, and final re-equilibration at 2% B for 5 min. The injection volume was 1 µL with a flow rate of 0.40 mL/min. The instrument settings of the mass spectrometer were as follows: sample cone, 30 V; the source temperature and desolvation temperature were 150°C and 450°C, respectively; the cone gas flow and desolvation gas flow were 50 and 600 L/h, respectively; and capillary voltages in negative-ion mode and in positive-ion mode were 2.5 and 3 kV, respectively. The mass range was set to 100 to 1,200 Da with a scan time of 0.35 s. The low collision energy was 6 eV and steadily increased from 15 to 30 eV, used as the high collision energy. A sodium formate solution (0.5 mM) was used to calibrate the instrument, and leucine enkephalin (200 pg/μL) was used as an external standard for mass correction during data collection. The MassLynx v.4.2 (Waters) was used to view mass spectra.

DESI-MSI was performed to analyze the spatial distribution of non-volatile components in *S. officinalis* leaves. The parameters referred to in the literature (Tong et al., 2022) are as follows: In both negative-ion mode and positive-ion mode, 98% methanol (v/v) and 100% methanol were used as solvent, capillary voltage was 3.5 kV in negative-ion mode and 4.5 kV in positive-ion mode with a flow rate was 2.5 µL/min, mass range was set from 100 to 1,200 Da, sampling cone was 60 V, collection angle and incident spray angle were respectively set as 10° and 65°, sprayer-to-inlet distance and sprayer-to-surface distance were 4 and 1 mm, respectively, and pixel resolution of images was 100 × 100 µm and viewed using HDI Imaging v1.4.

### 2.4 Data analysis for the volatile compounds

The calibration curves for retention time and retention index were established using butanone, 2-butanone, 2-pentanone, 2-hexanone, 2-heptanone, 2-octanone, and 2-nonanone. The chemical composition of volatile components was characterized using the NIST databases. The Laboratory Analytical Viewer was performed to plot the GC-MSI fingerprints of *S. miltiorrhiza* Bunge and *S. officinalis*. The Dynamic PCA software was used to perform principal component analysis (PCA).

### 2.5 Data analysis of LC-MS and DESI-MSI

The raw mass spectrum data were subjected to format conversion (ABF Converter, https://www.reifycs.com/AbfConverter/), and then the processed data were imported into MS-DIAL v 4.60 (http://prime.psc.riken.jp/compms/msdial/main.html) for peak detection, alignment, and normalization to obtain peak tables. The parameters were as follows: MS^1^ and MS^2^ tolerance was 0.01 and 0.02 Da, respectively; retention time range was 1–28 min; retention time tolerance was set to 0.15 min; MS^1^ and MS^2^ mass ranged from 100 to 5,000 Da; mass slice width was 0.1 Da; the minimum peak height was 20,000; and MS^2^ abundance cutoff was 800 amplitudes. Adduct types such as [M+H]^+^, [M+Na]^+^, [M+K]^+^, and [2M+H]^+^ were selected for positive-ion mode, and [M-H]^−^, [M+HCOO]^−^, [M+Na-2H]^−^, [M+K-2H]^−^, and [2M-H]^−^ were selected for negative-ion mode. The warfarin (5 μg/mL) in each data file was calculated in MS-DIAL for normalization processing.

### 2.6 Chemometric analysis

The normalized data were imported to SIMCA-P 14.1 (Umetrics AB, Umea, Sweden) to carry out the PCA. The differential metabolites between two species were screened by orthogonal partial least squares discriminate analysis (OPLS-DA), and compounds with variable importance in projection (VIP) of more than 1 were defined as potential chemical markers.

## 3 Results and discussion

### 3.1 Differential analysis of volatile components between *S. miltiorrhiza* Bunge and *S. officinalis*

**Figure 1:**
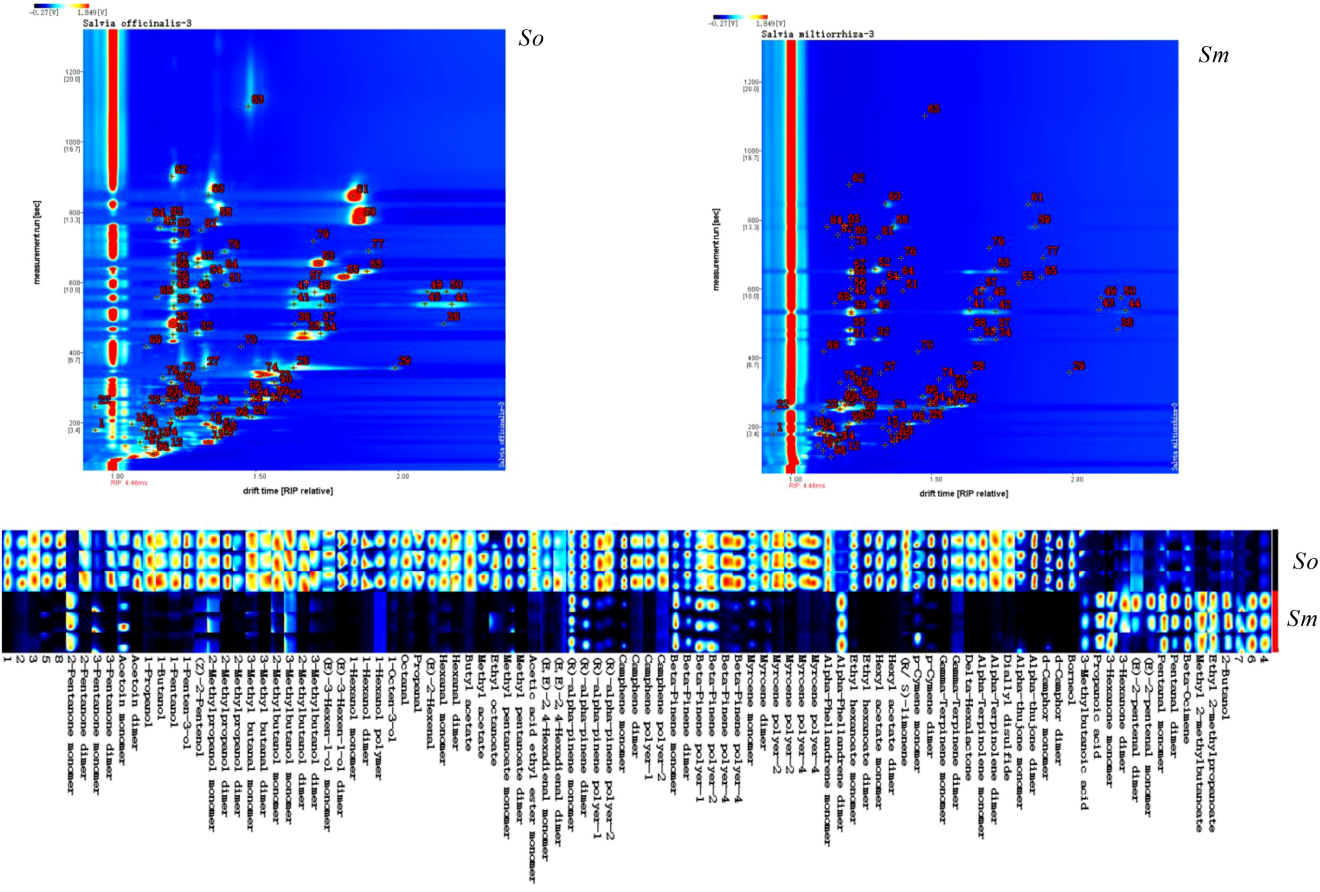
The volatile components detected from *S. officinalis* (*So*) *and S. miltiorrhiza* Bunge (*Sm*) using FlavourSpec^®^ and fingerprints of volatile components of *S. officinalis* (*So*) *and S. miltiorrhiza* Bunge (*Sm*).

FlavourSpec^®^ was carried out to analyze the distribution pattern of volatile components in the leaves of *S. miltiorrhiza* Bunge and *S. officinalis.* GC-IMS and the fingerprints of volatile components indicated the significant differences in the chemical composition of the two species (**Figure 1**). The red dots in the GC-IMS suggested the intensity of the ion signal; it can be seen that the content of volatile components in *S. officinalis* was generally higher than that in *S. miltiorrhiza* Bunge; therefore, it can be used to extract essential oils. *S. officinalis* can be widely used in perfume manufacturing and the development of health products due to the strong aroma derived from the high concentration of volatile components. The NIST database was then used to identify the chemical compositions of the volatile components. A total of 99 components were detected (**Figure 1**; **Supplementary Table 1**), and 8 compounds were not characterized among the 99 compounds.

**Figure 2:**
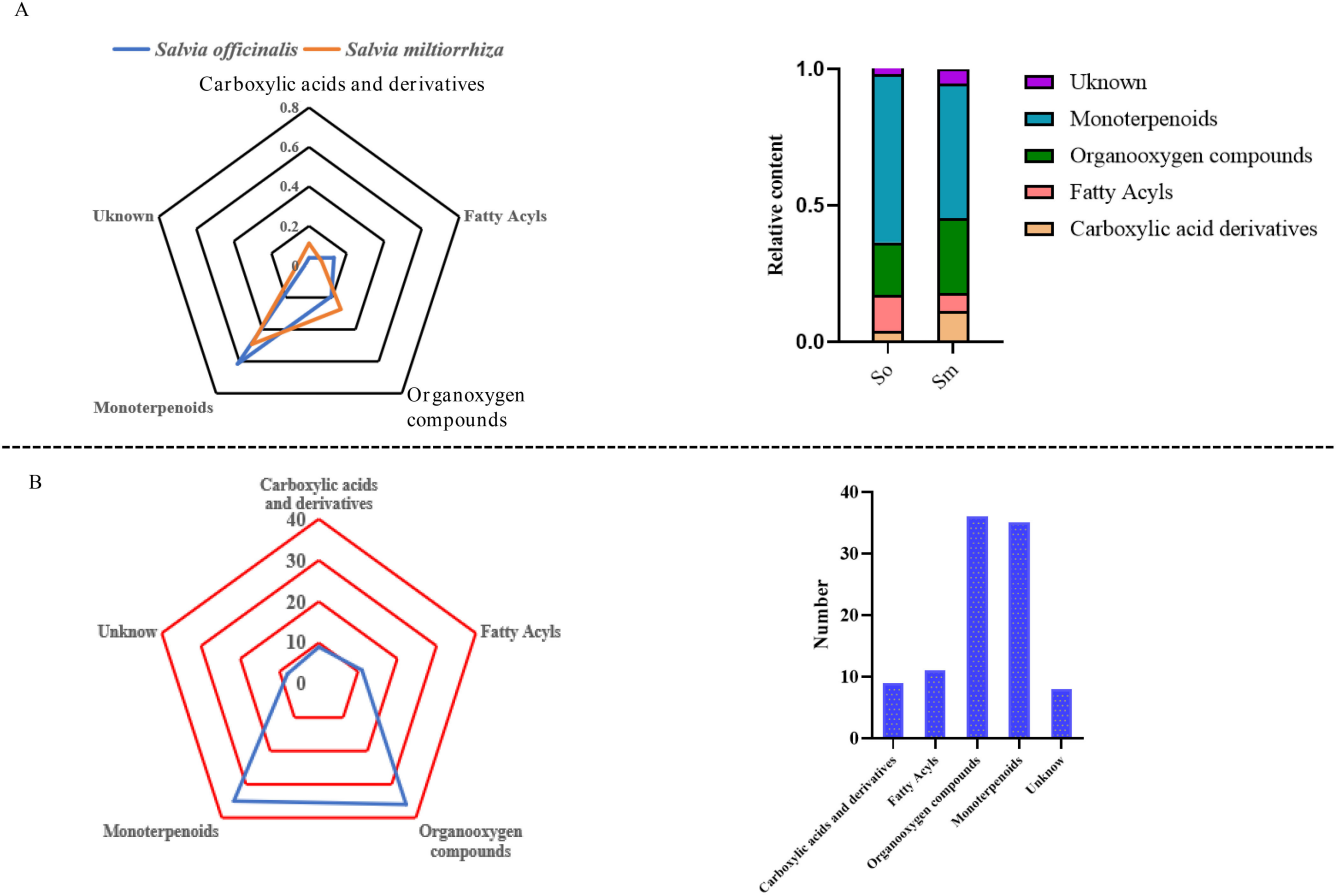
The relative content and number of different categories of volatile components in *S. officinalis* and *S. miltiorrhiza* Bunge. **(A)** The relative content of different categories of volatile components in *S. officinalis* and *S. miltiorrhiza* Bunge. **(B)** The number of different categories of volatile components in *S. officinalis* and *S. miltiorrhiza* Bunge.

PubChem (https://pubchem.ncbi.nlm.nih.gov/) and ClassyFire (http://classyfire.wishartlab.com/) were used to classify the volatile components. The results indicated that volatile components in the two important herbs were composed of monoterpenes, carboxylic acids and its derivatives, fatty acyls, and organooxygen compounds. The significant differences in the relative content and number of different categories of components are shown in **Figure 2**. The relative content of monoterpenes in the volatile components of *S. officinalis* was 0.616% and that in *S. miltiorrhiza* Bunge was 0.493%. Fatty acyls and carboxylic acid derivatives also had a high proportion in *S. officinalis* and *S. miltiorrhiza* Bunge, respectively. The relative content of fatty acyls in the volatile components of *S. officinalis* was four times higher than that in *S. officinalis* based on the findings of this work. The relative content of fatty acyls in *S. officinalis* was 0.313%, whereas that in *S. miltiorrhiza* Bunge was 0.067%. The carboxylic acid derivatives in the volatile components of *S. miltiorrhiza* Bunge were 0.112%, whereas those in *S. officinalis* were 0.039%. The proportion of several unidentified components in the volatile components of *S. miltiorrhiza* Bunge was significantly higher than that of *S. officinalis*, indicating that relatively less research was conducted on volatile components in *S. miltiorrhiza* Bunge. The organic oxygen compounds were the most abundant components identified among volatile compounds (36), followed by monoterpenoids, with 35 components identified as monoterpenoids (**Figure 2A**). The number of carboxylic acids and derivatives was nine. The number of these kinds of components was relatively low (**Figure 2B**). As an important aromatic medicinal plant, the volatile components of *S. officinalis* leaves have been extensively reported in literature. GC-MS was the mainstream method used for measuring volatile components. The results reported from the literature indicated that plant organic volatile small molecules mainly consisted of monoterpenes, sesquiterpenes, and their oxygen-containing compounds, fatty acid compounds (Baj et al., 2013). To the best of our knowledge, in this study, FlavourSpec^®^ was innovatively used to detect the volatile components of *S. officinalis* and *S. miltiorrhiza* Bunge. The results indicated that volatile components of *S. officinalis* and *S. miltiorrhiza* Bunge were mainly composed of monoterpenes and fatty acid compounds, consistent with previous literature reports (Doolaege et al., 2007). However, no compounds belonging to sesquiterpenes were detected. FlavourSpec^®^ was mainly used for detecting the odor of volatile components, indicating that the odor of *S. officinalis* leaves may mainly come from monoterpenes and their oxygen-containing derivatives.

**Figure 3:**
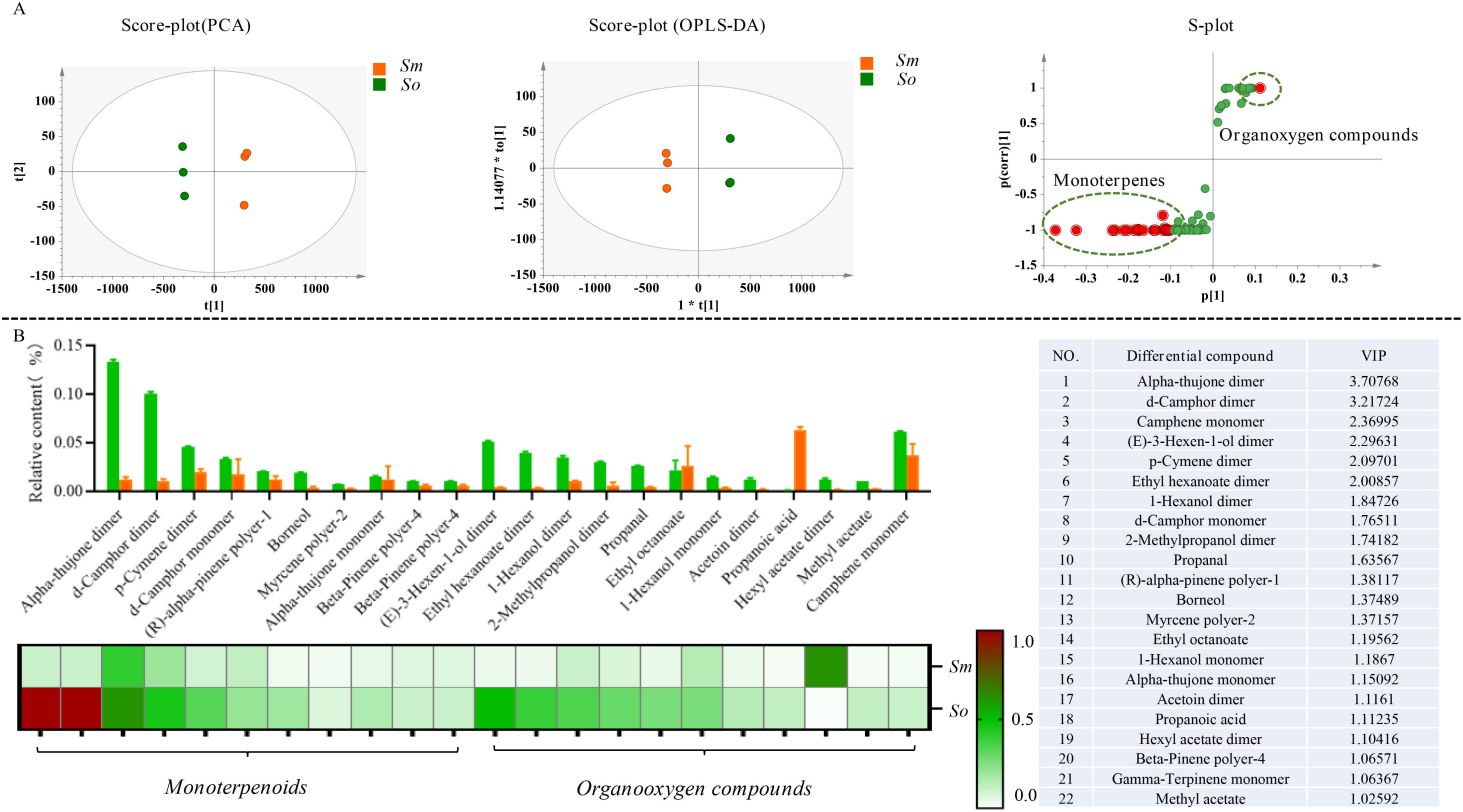
The significant difference in the volatile components was detected in *S. officinalis* and *S. miltiorrhiza* Bunge. **(A)** Multivariate statistical analysis of volatile components in *S. officinalis* and *S. miltiorrhiza* Bunge followed as PCA, OPLS-DA, and S-plot. **(B)** Distribution patterns of differential compounds in *S. officinalis* and *S. miltiorrhiza* Bunge.

Simca 14.0 was used to perform the PCA and OPLS-DA multivariate statistical analysis based on the distribution pattern of volatile components to explore the similarities and differences in the chemical compositions. The results indicated that *S. miltiorrhiza* Bunge and *S. officinalis* showed a significant trend of segregation based on PCA. *S. miltiorrhiza* Bunge and *S. officinalis* were grouped together separately. It was indicated that the chemical compositions of volatile components in the two medicinal species differed significantly (**Figure 3A**). In addition, it was found that the chemical composition of volatile components in *S. officinalis* was significantly richer than that in *S. miltiorrhiza* Bunge according to the fingerprint spectra of volatile components and showed significant differences in chemical composition. The OPLS-DA was performed to screen differential compounds, and a total of 22 compounds were regarded as significantly different compounds based on VIP > 1. The differential compounds were mainly composed of monoterpenes. It was indicated that the volatile components of aromatic plants mainly consist of monoterpenes, sesquiterpenes, and organic fatty acid compounds (**Figure 3B**). In this work, sesquiterpenes were almost not detected from the volatile components. Therefore, it was speculated that the odor of *S. officinalis* mainly comes from monoterpenes. The s-polt was used to show the distribution of differential compounds in two medicinal plants.

### 3.2 Analysis of differences in non-volatile components between *S. miltiorrhiza* Bunge and *S. officinalis*

**Figure 4:**
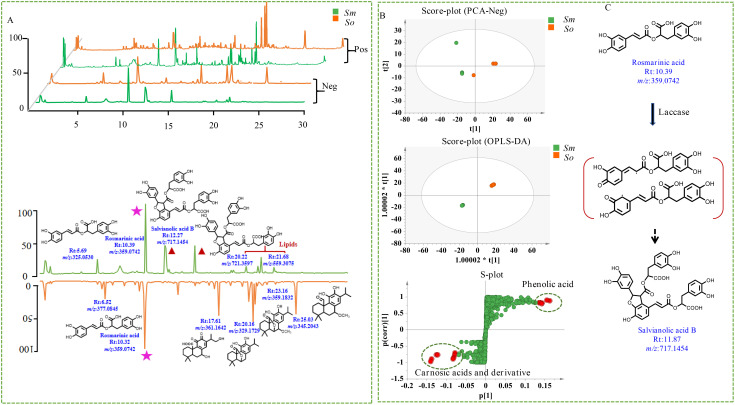
Multivariate statistical analysis of non-volatile components in *S. officinalis* and *S. miltiorrhiza* Bunge. **(A)** Total ion chromatogram of non-volatile components in *S. officinalis* and *S. miltiorrhiza* Bunge. **(B)** Chemical clustering analysis of *S. officinalis* and *S. miltiorrhiza* Bunge with different modeling methods. **(C)** Possible molecular mechanism of rosmarinic acid conversion to salvianolic acid B.

Tandem MS was performed to investigate the comprehensive metabolite profiles of *S. officinalis* and *S. miltiorrhiza* Bunge. It was performed in both positive- and negative-ion modes; the latter was more sensitive based on the total ion chromatogram. The differences and similarities in secondary metabolites between two herbs focused on the results in the negative-ion mode. The secondary metabolites in the leaves of *S. miltiorrhiza* were mainly concentrated within 18 min of elution, whereas secondary metabolites in the leaves of *S. officinalis* were mainly eluted within 25 min (**Figure 4A**). A total of 3,623 ions were detected, and MS-DIAL was performed to extract peak tables for the multivariate statistical analysis. The in-house database of *Salvia* genus was applied to characterize the chemicals, and 61 components were detected in the leaves of *S. officinalis* and *S. miltiorrhiza* Bunge, including organic acids, phenolic acids, and carnosic acid and its derivatives (**Supplementary Table 2**). The leaves of *S. officinalis* and *S. miltiorrhiza* were both detected with significant levels of rosmarinic acid. The content of danshensu and caffeic acid was relatively low, which was mainly used as a precursor for the synthesis of rosmarinic acid. It indicated the presence of active phenolic acid synthesis pathways in the leaves. Rosmarinic acid had significant antioxidant capacity and was important for leaves to prevent ultraviolet (UV) damage (Dahchour, 2022). Although the content of rosmarinic acid that was detected in the leaves was not significant, the content of rosmarinic acid in *S. miltiorrhiza* leaves was significantly higher than that in *S. officinalis.* The relative content of differential components in *S. miltiorrhiza* Bunge and *S. officinalis* is shown in **Figure 4A**. Salvianolic acid B, the significantly differential metabolite, was enriched with the leaves of *S. miltiorrhiza* but was almost undetectable in *S. officinalis.*

PCA was performed, and there was a clear separation trend indicating that the secondary metabolites in *S. miltiorrhiza* Bunge and *S. officinalis* were significantly different (**Figure 4B**). The OPLS-DA model was used to explore the differential compounds, and the compounds with a VIP of 4 were considered to be significantly differential compounds. A total of 20 structures were identified as differential compounds (**Supplementary Table 3**).

**Figure 5:**
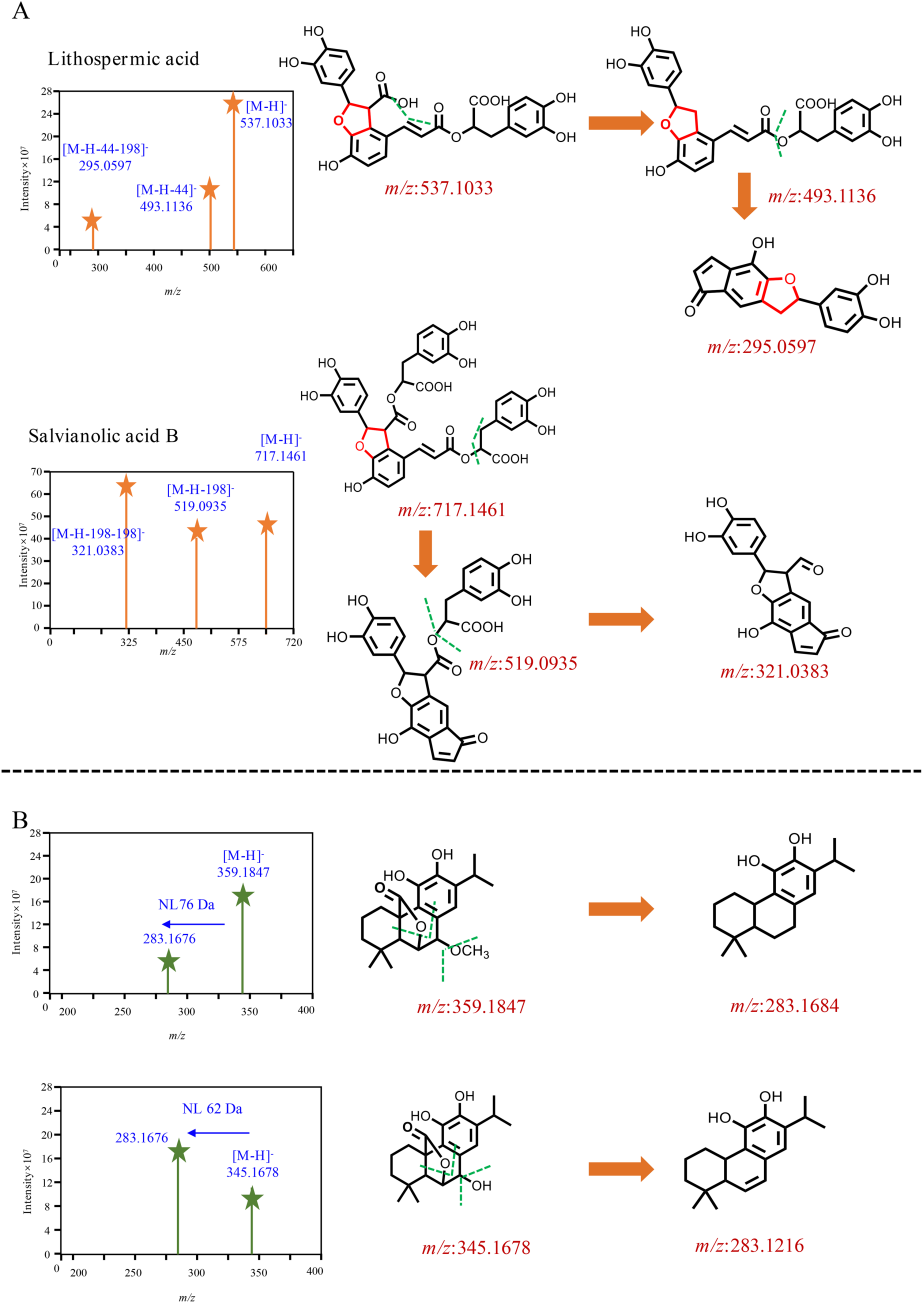
Mass spectrometry behavior of differential compounds. **(A)** The two components significantly enriched in *S. miltiorrhiza*, **(B)** The two components significantly enriched in *S. officinalis*.

The chemical characterization of differential compounds was performed based on the fragmentation pathway in MS. The main differential compounds along with the corresponding product ions are shown in **Figures 5A, B**. Take the differential compounds with a retention time of 21.16 and *m/z* 331.1917[M-H]^−^ as examples to explain structural characterization. The ESI source gave the [M-H-44]^−^ ions as the base peak, and the molecular formula was determined to be C_20_H_28_O_4_ according to [M-H]^−^. The MS/MS prominent ion was [M-H-44]^−^ at *m/z* 285.184 at 30% energy collision. The MS behavior and fragmentation pattern of neutral loss of the carboxyl group to produce fragment ions were consistent with those of carnosic acid reported in literature (Doolaege et al., 2007); thus, the ion was identified as carnosic acid. The retention of *m/z* 345.167[M-H]^−^ in the reverse-phase chromatography column was shorter than that of carnosic acid, indicating that the polarity was greater compared with carnosic acid (**Figure 5A**). The ions at *m/z* 345.167[M-H]^−^ was detected as base peak under the primary channel of mass spectrometry (MS^1^) (Doolaege et al., 2007). The two structures, as derivatives of carnosic acid, were mainly enriched in the leaves of *S. officinalis.* Two ions that significantly enriched leaves of *S. miltiorrhiza* Bunge were selected for chemical characterization, one ion at *m/z* 717.1417 [M-H]^−^, and another ion at *m/z* 537.1033 [M-H]^−^ (**Figure 5B**). The polarity of the two ions was greater than carnosic acid derivatives and can be eluted within 15 min. The molecular formula of the ion at *m/z* 717.1417 [M-H]^−^ was determined to be C_36_H_30_O_16_; the MS/MS ion was [M-H-198]^−^ at *m/z* 519 at 30% energy collision, suggesting its neutral loss of danshensu. The MS/MS prominent ion was [M-H-198-198]^−^ at *m/z* 321 at 30% energy collision, indicating that the loss of danshensu was easier than caffeic acid. The fragmentation pattern was consistent with salvianolic acid B that was reported in the literature (Liu et al., 2007). Similar to salvianolic acid B, the ion at *m/z* 537.1033 [M-H]^−^ was determined to be C_27_H_22_O_12_, and loss of the carboxyl group at *m/z* 493 [M-H-44]^−^ and danshensu yielded MS^2^ fragmentation ion at *m/z* 295 [M-H-44-198]^−^, which was identified as lithospermic acid (Liu et al., 2007). The two ions were significantly enriched in leaves of *S. miltiorrhiza* Bunge. It indicated that the differential compounds were mainly composed of phenolic acid and carnosic acid and its derivatives.

Previous studies have investigated the distribution of abietane-type diterpenes in the roots and leaves of both *S. miltiorrhiza* and *S. officinalis* (Li et al., 2020). The abietane-type diterpenes in *S. miltiorrhiza* roots were mainly composed of tanshinones, while the abietane-type diterpenes in *S. officinalis* were mainly enriched in the leaves, with carnosic acid and its derivatives as the main component. Tanshinones were not detected in either the leaves or roots of *S. officinalis* (Li et al., 2020). This work systematically investigated the distribution patterns of secondary metabolites in *S. miltiorrhiza* and *S. officinalis*, filling the gap in previous research and providing direction for the resource utilization and molecular breeding of the two species. For example, we found high levels of rosmarinic acid in the leaves of *S. officinalis*, indicating that the phenolic acid biosynthesis pathway was quite active in *S. officinalis* leaves. However, no components containing the furan structure such as salvianolic acid B were detected. According to previous research (Di et al., 2013), tetramer salvianolic acid B was mainly synthesized from rosmarinic acid as a substrate under the catalysis of laccase (**Figure 4C**). It was indicated that part of the rosmarinic acid synthesized from *S. miltiorrhiza* was used for downstream synthesis of salvianolic acid B, and another part was used for self-accumulation; thus, the content was significantly higher than that of *S. officinalis.* Previous research indicated that metabolites with a furan ring were the main active compounds of *S. miltiorrhiza*; in this work, salvianolic acid B was also found in the leaves. *S. officinalis* is considered an important medicinal and edible plant and is widely used in the treatment of neurodegenerative diseases and in condiments in Europe. In this work, the high content of rosmarinic acid without phenolic acid components containing furan structures was detected in this species. *S. officinalis* is now widely cultivated in various regions. Therefore, establishing a genetic transformation system for *S. officinalis* and utilizing biotechnology to cultivate new varieties of *S. officinalis* that can simultaneously synthesize polyphenolic acids such as salvianolic acid B and carnosic acid derivatives are important to the resource development of *Salvia* species. External environmental stress is the main driving force for enzyme evolution (Lau and Bolin, 2024); carnosic acid and its derivatives have been reported to have strong antioxidant capacity, which may be closely related to the adaptation of *S. officinalis* to harsh climates in the European Mediterranean region.

### 3.3 The spatial distribution of carnosic acid and its derivatives in *S. officinalis* leaves

**Figure 6:**
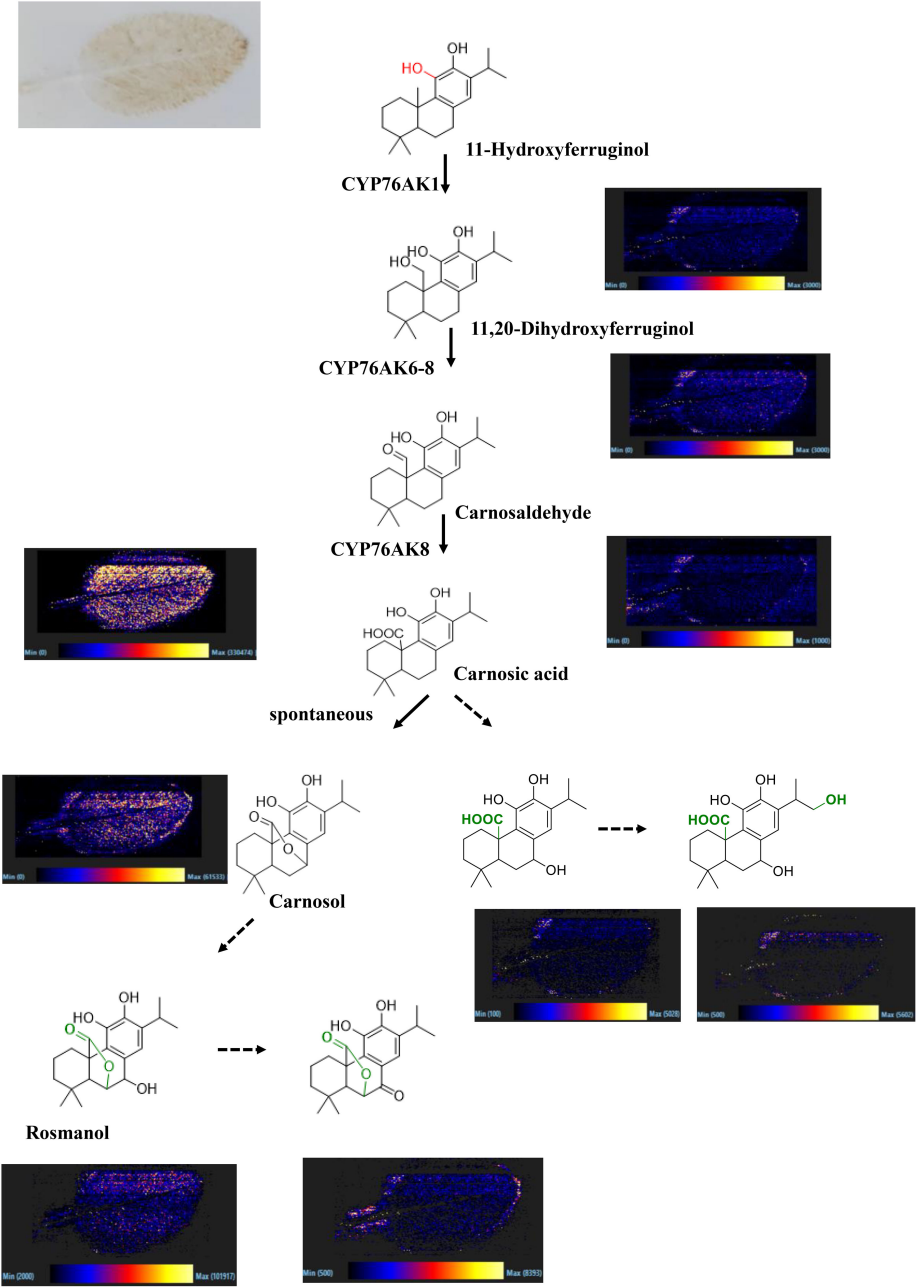
Spatial distribution of carnosic acid and its derivatives in the leaves of *S. officinalis*. **(A)** The two components significantly enriched in S. miltiorrhiza, **(B)** The two components significantly enriched in S. officinalis.

The spatial distribution of secondary metabolites in *S. miltiorrhiza* has been reported in the literature. However, the *in situ* distribution of carnosic acid and its derivatives in *S. officinalis* leaves is still unclear; thus, DESI was performed in this work to fill the research gap. The results indicated that carnosic acid and its derivatives showed wide distribution in the leaves (**Figure 6**); 11-hydroxyferruginol and 11,20-dihydroxyferruginol with a low content as precursors of carnosic acid and its derivatives only detected distribution at the leaves’ edge. Carnosic acid and its derivatives were reported with significant antioxidant effects (Birtić et al., 2015). *S. officinalis* was native to the Mediterranean region of Europe; the remarkable accumulation of carnosic acid and its derivatives is associated with the harsh climatic conditions. It was reported in the literature that carnosol was a spontaneous oxidation product of carnosic acid (Zhang et al., 2012). In this study, the two important metabolites were both found to be widely distributed in the leaves, indicating the relationship between carnosol and carnosic acid to some extent. Rosmanol was a structural analogue of carnosol that added a hydroxyl group at the C7 position and showed a weak ionic signal throughout the entire tissue range of the leaves. It suggested that rosmarinol and carnosol had an upstream–downstream relationship in their biosynthetic pathways. Spatial localization can also be used to analyze the biosynthetic pathways of secondary metabolites. The active compounds usually show a clear differential distribution in medicinal plants. For example, tanshinone was mainly distributed in the periderm of *S. miltiorrhiza* roots. Carnosic acid and its derivatives were widely distributed in the leaves of *S. officinalis*. The specific spatial distribution of secondary metabolites may be closely related to environmental pressure. Tanshinones have been reported to be secreted into the soil environment, inhibiting the growth of other plants and occupying ecological niches (Li et al., 2024), whereas the leaves were completely exposed to the environment and subjected to the UV radiation pressure caused by the wide distribution pattern of carnosic acid and its derivatives (Loussouarn et al., 2017).

## 4 Conclusion

The structure of secondary metabolites leads to the biological activity of medicinal plants. *S. miltiorrhiza* Bunge and *S. officinalis* are famous medicinal herbs widely used to treat cardiovascular and neurodegenerative diseases. The structural diversity of diterpenoids and their formation mechanisms in the roots have been reported. To explore the chemical diversity of secondary metabolites in the leaves, FlavourSpec^®^ combined with spatial metabolomics was conducted to analyze the accumulation patterns of metabolites that include volatile and non-volatile components. It was found that phenolic acid components such as rosmarinic acid and caffeic acid are widely distributed in *S. officinalis* and *S. miltiorrhiza* Bunge, and *S. miltiorrhiza* Bunge contained a high content of phenolic acid components with a furan ring structure; however, these kinds of compounds are hardly detected in *S. officinalis.* The compounds of *S. officinalis* leaves are mainly composed of carnosic acid and its derivatives, which exhibit widespread distribution characteristics in *S. officinalis* leaves. Therefore, it indicated that *S. officinalis* is an excellent raw material for extracting volatile components and developing functional foods due to its extensive antioxidant activity. In addition, the leaves of *S. officinalis* contained abundant volatile components compared with *S. miltiorrhiza* Bunge. This work analyzed the similarities and differences in secondary metabolites of *S. officinalis* and *S. miltiorrhiza* Bunge systematically, providing theoretical and material basis for the development and utilization of medicinal resources of *Salvia* spp.

## Data Availability

The datasets presented in this study can be found in online repositories. The names of the repository/repositories and accession number(s) can be found in the article/supplementary material.

## References

[B1] Baj T. Ludwiczuk A. Sieniawska E. Skalicka-Woźniak K. Widelski J. Zieba K.. (2013). GC-MS analysis of essential oils from *Salvia officinalis* L.: comparison of extraction methods of the volatile components. Acta Poloniae Pharm. 70, 35–40. 23610957

[B2] Beale D. J. Pinu F. R. Kouremenos K. A. Poojary M. M. Narayana V. K. Boughton B. A.. (2018). Review of recent developments in GC-MS approaches to metabolomics-based research. Metabolomics 14, 152. doi: 10.1007/s11306-018-1449-2 30830421

[B3] Birtić S. Dussort P. Pierre F. X. Bily A. C. Roller M. (2015). Carnosic acid. Phytochemistry 115, 9–19. doi: 10.1016/j.phytochem.2014.12.026 25639596

[B4] D’Auria M. Racioppi R. (2015). The effect of drying of the composition of volatile organic compounds in *Rosmarinus officinalis*, *Laurus nobilis*, *Salvia officinalis* and *Thymus serpyllum.* a hs-spme-gc-ms study. J. Essential Oil-Bearing Plants 18, 1209–1223. doi: 10.1080/0972060X.2014.895213

[B5] Dahchour A. (2022). Anxiolytic and antidepressive potentials of rosmarinic acid: A review with a focus on antioxidant and anti-inflammatory effects. Pharmacol. Res. 184, 106421. doi: 10.1016/j.phrs.2022.106421 36096427

[B6] Deng W. Chen F. Zhao Y. Zhou M. Guo M. (2023). Anti-hepatitis B virus activities of natural products and their antiviral mechanisms. Chin. J. Natural Medicines 21, 803–811. doi: 10.1016/S1875-5364(23)60505-9 38035936

[B7] Di P. Zhang L. Chen J. Tan H. Xiao Y. Dong X.. (2013). ¹³C tracer reveals phenolic acids biosynthesis in hairy root cultures of *Salvia miltiorrhiza* . ACS Chem. Biol. 8, 1537–1548. doi: 10.1021/cb3006962 23614461

[B8] Doolaege E. H. Raes K. Smet K. Andjelkovic M. Van Poucke C. De Smet S.. (2007). Characterization of two unknown compounds in methanol extracts of *Rosemary* oil. J. Agric. Food Chem. 55, 7283–7287. doi: 10.1021/jf071101k 17685542

[B9] Fiehn O. (2016). Metabolomics by gas chromatography-mass spectrometry: combined targeted and untargeted profiling. Curr. Protoc. Mol. Biol. 114, 30.4.1–30.4.32. doi: 10.1002/0471142727.mb3004s114 PMC4829120 27038389

[B10] Ghorbani A. Esmaeilizadeh M. (2017). Pharmacological properties of *Salvia officinalis* and its components. J. Traditional Complementary Med. 7, 433–440. doi: 10.1016/j.jtcme.2016.12.014 PMC5634728 29034191

[B11] Hu J. Qiu S. Wang F. Li Q. Xiang C. L. Di P.. (2023). Functional divergence of CYP76AKs shapes the chemodiversity of abietane-type diterpenoids in genus *Salvia* . Nat. Commun. 14, 4696. doi: 10.1038/s41467-023-40401-y 37542034 PMC10403556

[B12] Koubaa F. G. Abdennabi R. Salah A. S. B. El Feki A. (2019). Microwave extraction of *Salvia officinalis* essential oil and assessment of its GC-MS identification and protective effects versus vanadium-induced nephrotoxicity in Wistar rats models. Arch. Physiol. Biochem. 125, 404–413. doi: 10.1080/13813455.2018.1478427 29884068

[B13] Lau J. A. Bolin L. G. (2024). The tiny drivers behind plant ecology and evolution. Am. J. Bot. 111. doi: 10.1002/ajb2.16358 38666516

[B14] Li Y. Chen J. Zhi J. Huang D. Zhang Y. Zhang L.. (2024). The ABC transporter SmABCG1 mediates tanshinones export from the peridermic cells of Salvia miltiorrhiza root. J. Integr. Plant Biol. 67, 135-149. doi: 10.1111/jipb.13806 39575678

[B15] Li X. Wang Z. (2009). Chemical composition, antimicrobial and antioxidant activities of the essential oil in leaves of *Salvia miltiorrhiza* bunge. J. Essential Oil Res. 21, 476–480. doi: 10.1080/10412905.2009.9700222

[B16] Li S. Zhu N. Tang C. Duan H. Wang Y. Zhao G.. (2020). Differential distribution of characteristic constituents in root, stem and leaf tissues of *Salvia miltiorrhiza* using MALDI mass spectrometry imaging. Fitoterapia 146, 104679. doi: 10.1016/j.fitote.2020.104679 32619463

[B17] Liang Q. Liang Z. S. Wang J. R. Xu W. H. (2009). Essential oil composition of *Salvia miltiorrhiza* flower. Food Chem. 113, 592–594. doi: 10.1016/j.foodchem.2008.08.035

[B18] Liu J. Ge Z. Jiang X. Zhang J. Sun J. Mao X. (2023). A comprehensive review of natural products with anti-hypoxic activity. Chin. J. Natural Medicines 21, 499–515. doi: 10.1016/S1875-5364(23)60410-8 37517818

[B19] Liu A. H. Guo H. Ye M. Lin Y. H. Sun J. H. Xu M.. (2007). Detection, characterization and identification of phenolic acids in Danshen using high-performance liquid chromatography with diode array detection and electrospray ionization mass spectrometry. J. Chromatography. A 1161, 170–182. doi: 10.1016/j.chroma.2007.05.081 17574558

[B20] Lou J. Mao Z. Shan T. Wang Q. Zhou L. (2014). Chemical composition, antibacterial and antioxidant properties of the essential oils from the roots and cultures of *Salvia miltiorrhiza* . J. Essential Oil-Bearing Plants. 17, 380–384. doi: 10.1080/0972060x.2014.895199

[B21] Loussouarn M. Krieger-Liszkay A. Svilar L. Bily A. Birtić S. Havaux M. (2017). Carnosic Acid and Carnosol, Two major antioxidants of *rosemary*, act through different mechanisms. Plant Physiol. 175, 1381–1394. doi: 10.1104/pp.17.01183 28916593 PMC5664485

[B22] Ramakrishna A. Ravishankar G. A. (2011). Influence of abiotic stress signals on secondary metabolites in plants. Plant Signaling Behav. 6, 1720–1731. doi: 10.4161/psb.6.11.17613 PMC3329344 22041989

[B23] Tong Q. Zhang C. Tu Y. Chen J. Li Q. Zeng Z.. (2022). Biosynthesis-based spatial metabolome of *Salvia miltiorrhiza* Bunge by combining metabolomics approaches with mass spectrometry-imaging. Talanta 238, 123045. doi: 10.1016/j.talanta.2021.123045 34801902

[B24] Uţă G. Manolescu D. Ş. Avram S. (2021). Therapeutic properties of several chemical compounds of *Salvia officinalis* L. in Alzheimer’s Disease. Mini Rev. Medicinal Chem. 21, 1421–1430. doi: 10.2174/1389557521999201230200209 33390133

[B25] Wang Z. Peters R. J. (2022). Tanshinones: Leading the way into Lamiaceae labdane-related diterpenoid biosynthesis. Curr. Opin. Plant Biol. 66, 102189. doi: 10.1016/j.pbi.2022.102189 35196638 PMC8940693

[B26] Yang Y. Xie J. Wang Q. Wang L. Shang Y. Jiang Y.. (2024). Volatolomics-assisted characterization of the key odorants in green off-flavor black tea and their dynamic changes during processing. Food Chemistry: X 22, 101432. doi: 10.1016/j.fochx.2024.101432 38764783 PMC11101678

[B27] Zhan X. Qiu T. Zhang H. Hou K. Liang X. Chen C.. (2023). Mass spectrometry imaging and single-cell transcriptional profiling reveal the tissue-specific regulation of bioactive ingredient biosynthesis in *Taxus* leaves. Plant Commun. 4, 100630. doi: 10.1016/j.xplc.2023.100630 37231648 PMC10504593

[B28] Zhang J. Liang R. Wang L. Yang B. (2019). Effects and mechanisms of Danshen-Shanzha herb-pair for atherosclerosis treatment using network pharmacology and experimental pharmacology. J. Ethnopharmacology 229, 104–114. doi: 10.1016/j.jep.2018.10.004 30312741

[B29] Zhang Y. Smuts J. P. Dodbiba E. Rangarajan R. Lang J. C. Armstrong D. W. (2012). Degradation study of carnosic acid, carnosol, rosmarinic acid, and rosemary extract (*Rosmarinus officinalis* L.) assessed using HPLC. J. Agric. Food Chem. 60, 9305–9314. doi: 10.1021/jf302179c 22881034

