# Supplementary figures and images for: Biosynthesis-based metabolomics analysis reveals chemical diversity between two Salvia species
Source: Front Plant Sci. 2025 Jul 4;16:1613313. doi: 10.3389/fpls.2025.1613313 (PMC12271196; doi:10.3389/fpls.2025.1613313)

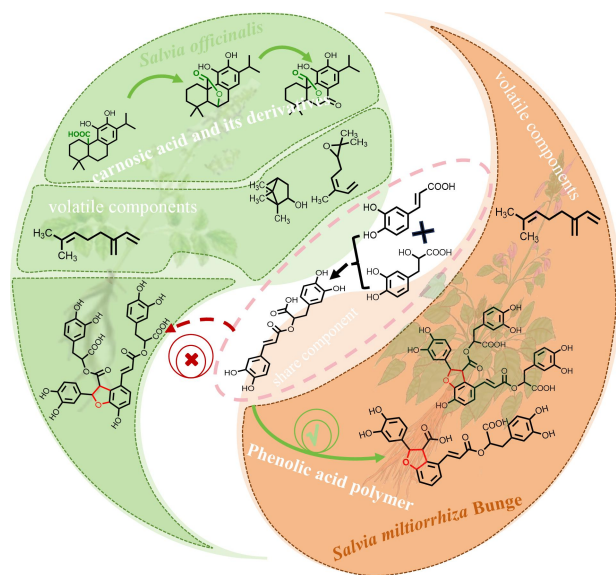

Supplement: Supplementary file 1 [file DataSheet1.pdf]
