# Supplementary material for: Biosynthesis-based metabolomics analysis reveals chemical diversity between two Salvia species
Source: Front Plant Sci. 2025 Jul 4;16:1613313. doi: 10.3389/fpls.2025.1613313 (PMC12271196; doi:10.3389/fpls.2025.1613313)
Supplement: Supplementary file 2 [file Table1.docx]

**Table S1 The volatile components identified from *S.miltiorrhiza* and *S.officinalis***

| **Compound** | **Molecular formula** | **Molecular Weight** | **Retention index** | **Retention time(s)** | **drift time (** a.u. **)** |
| --- | --- | --- | --- | --- | --- |
| 1-Penten-3-ol | C_5_H_10_O | 86.1 | 679.8 | 178.702 | 0.9391 |
| 3-Pentanone monomer | C_5_H_10_O | 86.1 | 697.6 | 189.244 | 1.1126 |
| 3-Pentanone dimer | C_5_H_10_O | 86.1 | 695.3 | 187.622 | 1.3511 |
| 2-Pentanone monomer | C_5_H_10_O | 86.1 | 678.1 | 177.891 | 1.1267 |
| 2-Pentanone dimer | C_5_H_10_O | 86.1 | 683.7 | 180.594 | 1.3782 |
| Propanoic acid | C_3_H_6_O_2_ | 74.1 | 682 | 179.783 | 1.1028 |
| 3-Methyl butanal monomer | C_5_H_10_O | 86.1 | 659.1 | 168.971 | 1.1787 |
| 3-Methyl butanal dimer | C_5_H_10_O | 86.1 | 659.6 | 169.241 | 1.4020 |
| 1-Butanol | C_4_H_10_O | 74.1 | 657.9 | 168.43 | 1.3749 |
| Acetic acid ethyl ester monomer | C_4_H_8_O_2_ | 88.1 | 599.3 | 143.832 | 1.0985 |
| Acetic acid ethyl ester dimer | C_4_H_8_O_2_ | 88.1 | 605.5 | 146.265 | 1.3359 |
| Methyl acetate | C_3_H_6_O_2_ | 74.1 | 558.5 | 123.829 | 1.1917 |
| 2-Butanol | C_4_H_10_O | 74.1 | 620.3 | 152.212 | 1.1451 |
| 2-Methylpropanol monomer | C_4_H_10_O | 74.1 | 627.5 | 155.185 | 1.1744 |
| 2-Methylpropanol dimer | C_4_H_10_O | 74.1 | 626.8 | 154.915 | 1.3673 |
| Acetoin monomer | C_4_H_8_O_2_ | 88.1 | 706.3 | 195.461 | 1.0692 |
| Acetoin dimer | C_4_H_8_O_2_ | 88.1 | 708.2 | 196.813 | 1.3272 |
| 2-Methylbutanol monomer | C_5_H_12_O | 88.1 | 737.3 | 219.249 | 1.2318 |
| 2-Methylbutanol dimer | C_5_H_12_O | 88.1 | 735.6 | 217.897 | 1.4757 |
| 3-Methylbutanol monomer | C_5_H_12_O | 88.1 | 731.6 | 214.653 | 1.2448 |
| 3-Methylbutanol dimer | C_5_H_12_O | 88.1 | 732.9 | 215.735 | 1.4898 |
| 2-Pentenol | C_5_H_10_O | 86.1 | 768.1 | 245.896 | 0.9405 |
| 2-pentenal monomer | C_5_H_8_O | 84.1 | 767.2 | 245.051 | 1.1157 |
| 2-pentenal dimer | C_5_H_8_O | 84.1 | 767.5 | 245.333 | 1.3563 |
| 3-Hexanone monomer | C_6_H_12_O | 100.2 | 773.6 | 250.961 | 1.1752 |
| 3-Hexanone dimer | C_6_H_12_O | 100.2 | 772.4 | 249.835 | 1.4707 |
| 1-Hexanol monomer | C_6_H_14_O | 102.2 | 871.1 | 355.503 | 1.3202 |
| 1-Hexanol dimer | C_6_H_14_O | 102.2 | 872 | 356.551 | 1.6350 |
| 1-Hexanol polymer | C_6_H_14_O | 102.2 | 872.3 | 356.901 | 1.9904 |
| Methyl-2-methylbutanoate | C_6_H_12_O_2_ | 116.2 | 776.1 | 253.25 | 1.5406 |
| alpha-pinene monomer | C_10_H_16_ | 136.2 | 934.2 | 450.866 | 1.2122 |
| alpha-pinene dimer | C_10_H_16_ | 136.2 | 936.7 | 455.34 | 1.2966 |
| alpha-pinene polyer-1 | C_10_H_16_ | 136.2 | 937.1 | 456.085 | 1.6734 |
| alpha-pinene polyer-2 | C_10_H_16_ | 136.2 | 936.3 | 454.594 | 1.7290 |
| Camphene monomer | C_10_H_16_ | 136.2 | 951.9 | 482.931 | 1.2122 |
| Camphene dimer | C_10_H_16_ | 136.2 | 951.1 | 481.44 | 1.6404 |
| Camphene polyer-1 | C_10_H_16_ | 136.2 | 951.1 | 481.44 | 1.7248 |
| Camphene polyer-2 | C_10_H_16_ | 136.2 | 951.5 | 482.185 | 2.1613 |
| Beta-Pinene monomer | C_10_H_16_ | 136.2 | 977.6 | 533.639 | 1.2163 |
| Beta-Pinene dimer | C_10_H_16_ | 136.2 | 978.3 | 535.13 | 1.2966 |
| Beta-Pinene polyer-1 | C_10_H_16_ | 136.2 | 979.8 | 538.113 | 1.6363 |
| Beta-Pinene polyer-2 | C_10_H_16_ | 136.2 | 978 | 534.385 | 1.7269 |
| Beta-Pinene polyer-3 | C_10_H_16_ | 136.2 | 980.1 | 538.859 | 2.0955 |
| Beta-Pinene polyer-4 | C_10_H_16_ | 136.2 | 979.8 | 538.113 | 2.1881 |
| Myrcene monomer | C_10_H_16_ | 136.2 | 997.2 | 575.399 | 1.2142 |
| Myrcene dimer | C_10_H_16_ | 136.2 | 997.2 | 575.399 | 1.2883 |
| Myrcene polyer-1 | C_10_H_16_ | 136.2 | 995.7 | 572.416 | 1.6363 |
| Myrcene polyer-2 | C_10_H_16_ | 136.2 | 995.4 | 571.67 | 1.7084 |
| Myrcene polyer-3 | C_10_H_16_ | 136.2 | 996 | 573.161 | 2.1016 |
| Myrcene polyer-4 | C_10_H_16_ | 136.2 | 996 | 573.161 | 2.1716 |
| Octanal | C_8_H_16_O | 128.2 | 1007.9 | 593.296 | 1.3975 |
| p-Cymene monomer | C_10_H_14_ | 134.2 | 1043.8 | 657.646 | 1.3002 |
| p-Cymene dimer | C_10_H_14_ | 134.2 | 1042.9 | 655.926 | 1.7235 |
| Ethyl hexanoate monomer | C_8_H_16_O_2_ | 144.2 | 1021.2 | 616.349 | 1.3298 |
| Ethyl hexanoate dimer | C_8_H_16_O_2_ | 144.2 | 1021.2 | 616.349 | 1.8085 |
| Alpha-Phellandrene monomer | C_10_H_16_ | 136.2 | 1012 | 600.289 | 1.2133 |
| Alpha-Phellandrene dimer | C_10_H_16_ | 136.2 | 1011.3 | 599.142 | 1.6791 |
| Alpha-thujone monomer | C_10_H_16_O | 152.2 | 1103.7 | 780.964 | 1.3649 |
| Alpha-thujone dimer | C_10_H_16_O | 152.2 | 1103.4 | 780.39 | 1.8677 |
| d-Camphor monomer | C_10_H_16_O | 152.2 | 1132.2 | 847.498 | 1.3372 |
| d-Camphor dimer | C_10_H_16_O | 152.2 | 1131.5 | 845.777 | 1.8436 |
| Borneol | C_10_H_18_O | 154.3 | 1154 | 902.164 | 1.2081 |
| Ethyl octanoate | C_10_H_20_O_2_ | 172.3 | 1223.5 | 1101.301 | 1.4773 |
| Hexyl acetate monomer | C_8_H_16_O_2_ | 144.2 | 1030.1 | 632.289 | 1.3855 |
| Hexyl acetate dimer | C_8_H_16_O_2_ | 144.2 | 1030.1 | 632.289 | 1.8918 |
| Limonene | C_10_H_16_ | 136.2 | 1030.1 | 632.289 | 1.2139 |
| Beta-Ocimene | C_10_H_16_ | 136.2 | 1041 | 652.385 | 1.2139 |
| 1-Octen-3-ol | C_8_H_16_O | 128.2 | 989.2 | 558.087 | 1.1571 |
| 2,4-Hexadienal monomer | C_6_H_8_O | 96.1 | 914.3 | 417.412 | 1.1193 |
| 2,4-Hexadienal dimer | C_6_H_8_O | 96.1 | 913.8 | 416.639 | 1.4525 |
| Methyl pentanoate monomer | C_6_H_12_O_2_ | 116.2 | 837.1 | 314.945 | 1.2065 |
| Methyl pentanoate dimer | C_6_H_12_O_2_ | 116.2 | 838.2 | 316.166 | 1.5669 |
| 3-Hexen-1-ol monomer | C_6_H_12_O | 100.2 | 857.7 | 338.947 | 1.2361 |
| 3-Hexen-1-ol dimer | C_6_H_12_O | 100.2 | 857.4 | 338.54 | 1.5258 |
| 2-Hexenal | C_6_H_10_O | 98.1 | 849.2 | 328.777 | 1.1769 |
| 1 | * | * | 1059.4 | 687.839 | 1.3931 |
| 2 | * | * | 1060.5 | 689.898 | 1.8964 |
| Gamma-Terpinene monomer | C_10_H_16_ | 136.2 | 1074.8 | 718.723 | 1.2177 |
| Gamma-Terpinene dimer | C_10_H_16_ | 136.2 | 1074.3 | 717.693 | 1.7051 |
| Alpha-Terpinolene monomer | C_10_H_16_ | 136.2 | 1089.8 | 750.456 | 1.2179 |
| Alpha-Terpinolene dimer | C_10_H_16_ | 136.2 | 1088.8 | 748.359 | 1.3124 |
| Delta-Hexalactone | C_6_H_10_O_2_ | 114.1 | 1092.4 | 756.048 | 1.1688 |
| Diallyl disulfide | C_6_H_10_S_2_ | 146.3 | 1104.1 | 781.91 | 1.1924 |
| 3 | * | * | 1102.9 | 779.114 | 1.1307 |
| Butyl acetate | C_6_H_12_O_2_ | 116.2 | 807.4 | 283.398 | 1.2395 |
| 4 | * | * | 811.5 | 287.546 | 1.4682 |
| 3-Methylbutanoic acid | C_5_H_10_O_2_ | 102.1 | 830.7 | 307.937 | 1.2232 |
| Hexanal monomer | C_6_H_12_O | 100.2 | 797.6 | 273.721 | 1.2580 |
| Hexanal dimer | C_6_H_12_O | 100.2 | 795.1 | 271.301 | 1.5651 |
| 5 | * | * | 826.6 | 303.444 | 1.5749 |
| Ethyl 2-methylpropanoate | C_6_H_12_O_2_ | 116.2 | 787.1 | 263.698 | 1.1949 |
| 6 | * | * | 785.2 | 261.97 | 1.6075 |
| 7 | * | * | 794.7 | 270.956 | 1.1785 |
| 8 | * | * | 791.1 | 267.5 | 1.4954 |
| 1-Pentanol | C_5_H_12_O | 88.1 | 762.6 | 240.887 | 1.2526 |
| Propanal | C_3_H_6_O | 58.1 | 533.5 | 112.106 | 1.1432 |
| 1-Propanol | C_3_H_8_O | 60.1 | 572.2 | 130.762 | 1.1158 |
| Pentanal monomer | C_5_H_10_O | 86.1 | 726.1 | 210.37 | 1.2054 |
| Pentanal dimer | C_5_H_10_O | 86.1 | 727.1 | 211.123 | 1.4211 |

Note: * means the information was unknown.

**Table S2 The secondary metabolites identified from *S.miltiorrhiza* and *S.officinalis***

| **No.** | **Retention time（min）** | **Measured [M-H]^-^ (*m/z*)** | **Molecular**  **formula** | **Error (ppm)** | **Identification** |
| --- | --- | --- | --- | --- | --- |
| 1 | 1.63 | 167.0337 | C_8_H_8_O_4_ | -8.2 | 2-Hydroxy-3',4'-dihydroxyacetophenone |
| 2 | 3.280 | 197.0431 | C_9_H_10_O_5_ | -4.5 | Danshensu |
| 3 | 5.800 | 179.0326 | C_9_H_8_O_4_ | -0.8 | Caffeic acid |
| 4 | 5.800 | 325.054 | C_14_H_14_O_9_ | 2.1 | Fertaric acid |
| 5 | 6.525 | 377.0845 | C_18_H_18_O_9_ | -1.5 | Salvianic acid C/isomer |
| 6 | 7.047 | 537.1011 | C_27_H_22_O_12_ | -0.8 | Salvianolic acid J |
| 7 | 7.100 | 137.0235 | C_7_H_6_O_3_ | -13 | Protocatechuic aldehyde |
| 8 | 7.820 | 179.0338 | C_9_H_8_O_4_ | -6.3 | Caffeic acid isomer |
| 9 | 7.992 | 539.1127 | C_27_H_24_O_12_ | -1.3 | Unknown |
| 10 | 8.152 | 377.0871 | C_18_H_18_O_9_ | -1.9 | Salvianic acid C/isomer |
| 11 | 9.037 | 735.1532 | C_36_H_32_O_17_ | 0.8 | 8-Hydroxy-salvianolic acid B/isomer |
| 12 | 9.138 | 537.1001 | C_27_H_22_O_12_ | -1.1 | Salvianolic acid H |
| 13 | 9.138 | 537.0996 | C_27_H_22_O_12_ | -1.4 | Lithospermic acid |
| 14 | 9.138 | 537.0996 | C_27_H_22_O_12_ | 1.2 | Lithospermic acid/isomer |
| 15 | 9.290 | 359.0774 | C_18_H_16_O_8_ | -0.4 | Rosmarinic acid |
| 16 | 9.310 | 537.1046 | C_27_H_22_O_12_ | 1.3 | Lithospermic acid isomer |
| 17 | 9.982 | 879.1978 | C_42_H_40_O_21_ | 0 | Salvianolic acid B 4-O-glucoside/isomer |
| 18 | 10.445 | 313.072 | C_17_H_14_O_6_ | -0.6 | Salvianolic acid F |
| 19 | 11.288 | 539.1169 | C_27_H_24_O_12_ | 0.1 | Unknown |
| 20 | 12.270 | 715.1246 | C_36_H_28_O_16_ | -0.5 | 7,8-Didehydro-salcianolic acid B/isomer |
| 21 | 12.270 | 717.145 | C_36_H_30_O_16_ | -0.4 | Salvianolic acid L |
| 22 | 12.27 | 717.1418 | C_36_H_30_O_16_ | -6 | Salvianolic acid B |
| 23 | 12.334 | 313.069 | C_17_H_14_O_6_ | 1.9 | Salvianolic acid F isomer |
| 24 | 12.338 | 717.1403 | C_36_H_30_O_16_ | -0.4 | Salvianolic acid B isomer |
| 25 | 12.590 | 747.1505 | C_37_H_32_O_17_ | 0.6 | 5-Hydroxyl-9-Methyl salvianolic acid B/isomer |
| 26 | 12.595 | 747.1507 | C_37_H_32_O_17_ | -0.1 | 5-Hydroxyl-9-Methyl salvianolic acid B/isomer |
| 27 | 12.670 | 537.1047 | C_27_H_22_O_12_ | 1.5 | Lithospermic acid/isomer |
| 28 | 12.957 | 539.1163 | C_27_H_24_O_12_ | 1.3 | Unknown |
| 29 | 13.219 | 735.1524 | C_36_H_32_O_17_ | -1.1 | 8-Hydroxy-salvianolic acid B/isomer |
| 30 | 13.640 | 373.0915 | C_19_H_18_O_8_ | 1.1 | Methyl rosmarinate |
| 31 | 13.741 | 701.1458 | C_36_H_30_O_15_ | -0.7 | 3-Deoxy-salvianolicacid B/isomer |
| 32 | 13.741 | 701.1458 | C_36_H_30_O_15_ | 0.2 | 3-Deoxy-salvianolicacid B/isomer |
| 33 | 13.902 | 551.1192 | C_28_H_24_O_12_ | -0.6 | 9-Methyl lithospermate/isomer |
| 34 | 13.902 | 731.1586 | C_37_H_32_O_16_ | 1.1 | 9-Methyl lithospermate B |
| 35 | 14.686 | 685.1542 | C_36_H_30_O_14_ | 0.8 | 3-Deoxy-3-deoxy- salvianolic acid B |
| 36 | 14.880 | 731.1573 | C_37_H_32_O_16_ | -0.8 | 9-Methyl salvianolic acid B |
| 37 | 15.208 | 717.1459 | C_36_H_30_O_16_ | -0.3 | Salvianolic acid E |
| 38 | 15.209 | 747.1577 | C_37_H_32_O_17_ | 1.4 | 5-Hydroxyl-9-Methyl salvianolic acid B/isomer |
| 39 | 15.209 | 313.0684 | C_17_H_14_O_6_ | 3 | Salvianolic acid F isomer |
| 40 | 15.310 | 717.1469 | C_36_H_30_O_16_ | -0.7 | Salvianolic acid B isomer |
| 41 | 15.310 | 717.1469 | C_36_H_30_O_16_ | 8.6 | Salvianolic acid B isomer |
| 42 | 15.410 | 551.1158 | C_28_H_24_O_12_ | 2.1 | 9-Methyl lithospermate/isomer |
| 43 | 15.832 | 491.0974 | C_26_H_20_O_10_ | 2.8 | Salvianolic acid C |
| 44 | 16.350 | 701.1512 | C_36_H_30_O_15_ | 0 | 3-Deoxy-salvianolicacid B/isomer |
| 45 | 16.355 | 701.1512 | C_36_H_30_O_15_ | 0 | 3-Deoxy-salvianolicacid B/isomer |
| 46 | 16.750 | 331.1914 | C_20_H_28_O_4_ | 1.5 | Carnosic acid |
| 47 | 17.677 | 345.1701 | C_20_H_26_O_5_ | -0.3 | Isorosmarinol |
| 48 | 18.285 | 345.1672 | C_20_H_26_O_5_ | -0.3 | Rosmarinol |
| 49 | 20.435 | 343.1539 | C_20_H_24_O_5_ | -1.7 | 7-Hydroxyisocarnosol |
| 50 | 20.530 | 343.1539 | C_20_H_24_O_5_ | -1.7 | 6-Hydroxycarnosol |
| 51 | 20.898 | 359.1832 | C_21_H_28_O_5_ | -2.5 | Unknown |
| 52 | 20.950 | 361.1642 | C_20_H_26_O_6_ | -1.9 | Unknown |
| 53 | 21.060 | 329.1749 | C_20_H_26_O_4_ | -1.2 | Carnosol |
| 54 | 22.264 | 315.1936 | C_20_H_28_O_3_ | -2.2 | 7-Carbonyl, 11-hydroxyferruginol |
| 55 | 24.978 | 317.2112 | C_20_H_30_O_3_ | -2.2 | 6, 11-Dihydroxyferruginol |
| 56 | 25.079 | 345.2043 | C_21_H_30_O_4_ | -2.3 | Unknown |
| 57 | 25.080 | 301.2146 | C_20_H_30_O_2_ | -2.7 | 11-Hydroxyferruginol |
| 58 | 25.080 | 317.2095 | C_20_H_30_O_3_ | -2.2 | 11, 20-Dihydroxyferruginol |
| 59 | 26.910 | 317.2115 | C_20_H_30_O_3_ | -0.6 | 7, 11-Dihydroxyferruginol |
| 60 | 28.477 | 299.1989 | C_20_H_28_O_2_ | -0.3 | 7-Carbonyl-ferruginol |

**Table S3 The differential compounds identified from *S. miltiorrhiza and S. officinalis***

| **No.** | **Retention time** | **Molecular weight** | **VIP** | **Differential Component** | **Molecular formula** |
| --- | --- | --- | --- | --- | --- |
| 1 | 25.139 | 345.203 | 9.521 | 7-Methyl-carnosic acid | C20H26O5 |
| 2 | 10.344 | 719.1569 | 9.121 | Rosmarinic acid dimer | C40H56O8 |
| 3 | 20.536 | 343.1521 | 8.898 | 7-Carbonyl-carnosol | C20H24O5 |
| 4 | 16.75 | 331.1914 | 8.326 | Carnosic acid | C20H28O4 |
| 5 | 17.661 | 345.1701 | 8.317 | Isorosmarinol | C20H26O5 |
| 6 | 12.274 | 717.1418 | 7.130 | Salvianic acid B | C36H30O16 |
| 7 | 10.344 | 741.1384 | 7.057 | Rosmarinic acid dimer+Na | C40H56O8 |
| 8 | 6.525 | 377.0845 | 6.636 | Salvianic acid C/isomer | C26H20O10 |
| 9 | 16.75 | 397.16 | 5.559 | Carnosic acid+COOH | C20H28O4 |
| 10 | 15.208 | 717.1459 | 5.494 | Salvianic acid B isomer | C36H30O16 |
| 11 | 18.285 | 345.1672 | 4.919 | Rosmarinol | C20H26O5 |
| 12 | 12.274 | 739.1229 | 4.154 | Salvianic acid B+Na | C36H30O16 |

Note: VIP means variable importance in projection
